# Multicenter cohort study on the presentation and treatment of acute appendicitis during the COVID-19 pandemic

**DOI:** 10.1007/s00384-022-04137-3

**Published:** 2022-04-12

**Authors:** Demi Huijgen, Elisabeth M. L. de Wijkerslooth, Josephine C. Janssen, Frédérique H. Beverdam, Evert-Jan G. Boerma, Jan Willem T. Dekker, Sophia Kitonga, Charles C. van Rossem, Wilhelmina H. Schreurs, Boudewijn R. Toorenvliet, Maarten Vermaas, Bas P. L. Wijnhoven, Anne Loes van den Boom

**Affiliations:** 1 Department of Surgery, Erasmus MC – University Medical Center, PO Box 2040, 3000 CA Rotterdam, the Netherlands; 2 Department of Surgery, Franciscus Hospital & Vlietland, Rotterdam, The Netherlands; 3 Department of Surgery, Zuyderland Medical Center, Heerlen, The Netherlands; 4 Department of Surgery, Reinier de Graaf Hospital, Delft, The Netherlands; 5 Department of Surgery, Medical Center Leeuwarden, Leeuwarden, The Netherlands; 6 Department of Surgery, Maasstad Hospital, Rotterdam, The Netherlands; 7 Department of Surgery, Northwest Clinics, Alkmaar, The Netherlands; 8 Department of Surgery, Ikazia Hospital, Rotterdam, The Netherlands; 9 Department of Surgery, IJsselland Hospital, Capelle Aan Den IJssel, The Netherlands

**Keywords:** Appendicitis, Appendectomy, Surgery, COVID-19

## Abstract

**Purpose:**

Current studies have demonstrated conflicting results regarding surgical care for acute appendicitis during the COVID-19 pandemic. This study aimed to assess trends in diagnosis as well as treatment of acute appendicitis in the Netherlands during the first and second COVID-19 infection wave.

**Methods:**

All consecutive patients that had an appendectomy for acute appendicitis in nine hospitals from January 2019 to December 2020 were included. The primary outcome was the number of appendectomies for acute appendicitis. Secondary outcomes included time between onset of symptoms and hospital admission, proportion of complex appendicitis, postoperative length of stay and postoperative infectious complications. Outcomes were compared between the pre-COVID group and COVID group.

**Results:**

A total of 4401 patients were included. The mean weekly rate of appendectomies during the COVID period was 44.0, compared to 40.9 in the pre-COVID period. The proportion of patients with complex appendicitis and mean postoperative length of stay in days were similar in the pre-COVID and COVID group (respectively 35.5% vs 36.8%, *p* = 0.36 and 2.0 ± 2.2 vs 2.0 ± 2.6, *p* = 0.93). There were no differences in postoperative infectious complications. A computed tomography scan was used more frequently as a diagnostic tool after the onset of COVID-19 compared to pre-COVID (13.8% vs 9.8%, *p* < 0.001, respectively).

**Conclusion:**

No differences were observed in number of appendectomies, proportion of complex appendicitis, postoperative length of stay or postoperative infectious complications before and during the COVID-19 pandemic. A CT scan was used more frequently during the COVID-19 pandemic.

## Introduction

Coronavirus disease 2019 (COVID-19) rapidly became a global pandemic late 2019 [1]. The first confirmed case of severe acute respiratory syndrome coronavirus-2 (SARS-CoV-2) infection in the Netherlands dates back to February 27, 2020 [2]. In an attempt to slow down the spread of the virus, the government introduced a nationwide lockdown from March 12, 2020 [2]. Due to an increasing number of COVID-19 admissions, standard hospital care around the world was disrupted [3]. In many Dutch hospitals, elective surgical care was downscaled [4]. Hospital referrals by general practitioners decreased and patients avoided hospital care due to the fear of becoming infected or with the intention of not burdening the healthcare system [4].

Considering that acute abdominal pain, including appendicitis, is a condition that requires emergency (surgical) care, one would not expect a decrease in hospital admissions during the COVID-19 pandemic, yet multiple studies have demonstrated a significant decrease in referrals for acute appendicitis [5–9]. In addition, several studies have reported an increase in the proportion of complex acute appendicitis [6, 8–16], as well as a prolonged time between onset of symptoms and hospital admission [8, 10–12, 14].

In response to the many COVID-19 admissions and shortage of hospital resources, non-operative management of (suspected) simple acute appendicitis was implemented on a wider scale in some regions [17, 18]. Furthermore, open appendectomy was advised by some organizations as a way of avoiding the potential risk of aerosolization during laparoscopy [17, 19]. The Dutch Association of Surgeons did not make specific recommendations for the treatment of acute appendicitis during the COVID-19 pandemic [20]. On a local level however, Dutch surgeons may have adjusted their approach. As of yet, no large-scale analysis has been published on the impact of the first as well as the second wave of COVID-19 infections and subsequent lockdowns on the presentation and treatment of acute appendicitis in the Netherlands.

We hypothesized that the pandemic may have resulted in an increased time between onset of symptoms and hospital admission, a reduced number of appendectomies for acute appendicitis, an increased proportion of complex appendicitis, and a higher post-operative complication rate.

## Materials and methods

### Study design

A multicenter retrospective observational cohort study was conducted. All consecutive patients with a registered appendectomy for acute appendicitis in one of nine Dutch hospitals (one academic center and eight teaching hospitals) were eligible for inclusion.

### Participants

All patients who had an appendectomy from January 2019 through December 2020 were selected. Eligible patients were identified from the hospitals’ electronic databases by selecting procedure codes for open and laparoscopic appendectomy (used in Dutch healthcare reimbursement). Patients were excluded in case of an appendectomy indication other than acute appendicitis (interval/delayed appendectomy, incidental appendectomy (as part of a larger surgical procedure), elective appendectomy due to other (suspected) pathology (appendiceal neoplasm or mucocele), or in case of appendix sana).

### Time periods

**Fig. 1:**
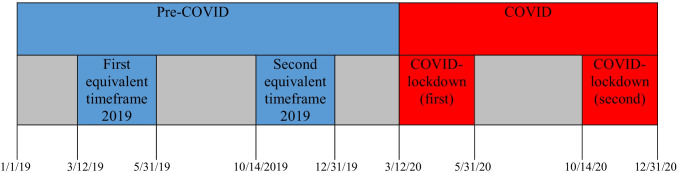
Overview of the cohort timeframes

Patients were categorized according to the date of COVID lockdown in the Netherlands. The pre-COVID group included patients undergoing appendectomy between January 1, 2019, and March 11, 2020, whereas the COVID group included patients that underwent appendectomy between March 12, 2020, and December 31, 2020. In the Netherlands, two lockdown periods took place. Therefore, the COVID group was further subdivided into the first COVID-lockdown group (March 12, 2020, to May 31^st^, 2020) and the second COVID-lockdown group (October 14, 2020, to December 31, 2020). Patients from these two time periods were compared with patients that underwent appendectomy during equivalent timeframes in 2019 (Fig. 1).

### Outcomes and data collection

The primary outcome was number of appendectomies for acute appendicitis per specified timeframe. Secondary outcomes were time between onset of symptoms and hospital admission (in days), proportion of intra-operative diagnosis of complex appendicitis, postoperative length of stay (in days) and postoperative infectious complications. Complex appendicitis was defined as intraoperative findings of necrosis, perforation, abscess and/or diffuse peritonitis [21]. Postoperative infectious complications were defined as an intra-abdominal abscess (IAA) or a surgical site infection (SSI) in the postoperative course, within 90 days after appendectomy, classified according to the Clavien-Dindo classification [22]. All data was extracted from electronic patient files and included baseline patient characteristics (age, sex, body mass index (BMI), American Society of Anesthesiologists (ASA) score), preoperative parameters (date of onset of symptoms, body temperature, serum C-reactive protein (CRP), white blood cell count (WBC)), imaging (ultrasound (US), computed tomography (CT) or multiple imaging studies including MRI scan), admission details (date and time of admission to hospital, date and time of discharge from hospital), intra-operative parameters (date and time of surgery, surgical approach, intra-operative findings, duration of surgery), postoperative outcomes (postoperative infectious complications like IAA and SSI, postoperative antibiotic treatment and duration, hospital readmission), and COVID-19 diagnostics (chest X-ray, chest CT, PCR test, or COVID antibody serology).

### Statistical analysis

Statistical analysis was performed in SPSS (IBM SPSS Statistics for Windows, version 26.0. Released 2019. Armonk, NY: IBM Corp). Outcomes were compared between the groups using the independent Student’s *t* test in case of continuous outcome variables and the Chi-square test in case of categorical outcome variables. The level of statistical significance was set at *p* < 0.05.

## Results

### Study population

**Fig. 2:**
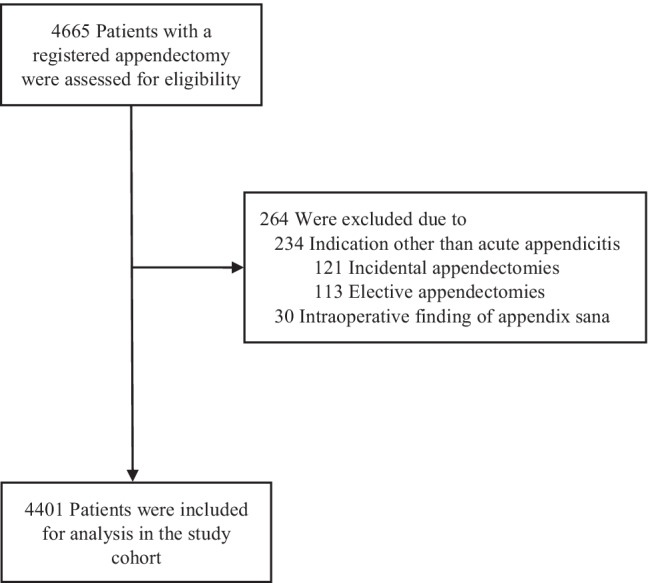
Cohort selection flowchart

During the study period, 4665 patients with a hospital registry code for open or laparoscopic appendectomy were identified and 264 patients were excluded (Fig. 2). No statistically significant differences were found in baseline characteristics between patients in the pre-COVID (*n* = 2547) and COVID group (*n* = 1854) (Table 1). Table 2 shows patient characteristics for the two COVID-lockdown groups compared to patients that underwent appendectomy in 2019.

**Table 1:** Comparison of patient characteristics between the pre-COVID and COVID group

	**COVID**^a^ **(*n* = 1854)**	**Pre-COVID**^b^ **(*n* = 2547)**	***p* value**
Age, mean ± *SD*	36.7 ± 20.6	36.6 ± 20.5	0.896
Male sex, *n* (%)	937 (50.6)	1262 (49.5)	0.505
BMI, mean ± *SD*	24.5 ± 5.7	24.8 ± 5.4	0.135
ASA class ≥ III, *n* (%)	138 (7.8)	195 (7.8)	0.984
Body temperature ≥ 38 °C, *n* (%)	355 (19.1)	554 (21.8)	**0.035**
CRP, mg/L, mean ± *SD*	71.9 ± 77.8	72.6 ± 79.1	0.781
WBC ≥ 11 × 10^9^/L, *n* (%)	1409 (76.0)	1883 (73.9)	0.119
Diagnostic test, *n* (%)			
US	1181 (63.7)	1689 (66.3)	0.072
CT	256 (13.8)	249 (9.8)	** < 0.001**
Multiple imaging studies	403 (21.7)	575 (22.6)	0.509
Duration of abdominal pain > 1 day, *n* (%)	702 (37.9)	1033 (40.6)	0.071
Total length of stay, days, mean ± *SD*	2.3 ± 2.6	2.3 ± 2.3	0.830
Postoperative length of stay, days, mean ± *SD*	2.0 ± 2.6	2.0 ± 2.2	0.933
Laparoscopic surgery, *n* (%)	1807 (97.6)	2450 (96.3)	**0.018**
Duration of surgery, minutes, mean ± *SD*	42.2 ± 18.0	41.2 ± 18.0	0.082
Complex appendicitis, *n* (%)	682 (36.8)	900 (35.5)	0.363
Antibiotics use, *n* (%)	664 (35.8)	901 (35.4)	0.764
Duration of antibiotics use, days, mean ± *SD*	5.0 ± 3.0	4.7 ± 2.8	0.083
Postoperative complications, *n* (%)			
IAA	60 (3.2)	99 (3.9)	0.253
SSI	35 (1.9)	51 (2.0)	0.786
Clavien-Dindo classification ≥ 3, *n* (%)	34 (37.4)	59 (40.1)	0.670
Readmissions, *n* (%)	95 (5.1)	144 (5.7)	0.445

^a^COVID: March 12, 2020, to December 31, 2020

^b^Pre-COVID: January 1, 2019, to March 11, 2020

Numbers may not total 4401 due to missing data

**Table 2:** Comparison of patient characteristics between the first and second lockdown and corresponding timeframes of 2019

	**First** **COVID-lockdown**^a^ **(*n* = 474)**	**First** **corresponding timeframe 2019**^b^ **(*n* = 470)**	***p* value**	**Second** **COVID-** **Lockdown**^c^ **(*n* = 495)**	**Second** **corresponding timeframe 2019**^d^ **(*n* = 448)**	***p* value**
Age, mean ± *SD*	35.7 ± 20.9	37.1 ± 21.5	0.317	38.1 ± 20.8	34.7 ± 19.5	**0.010**
Male sex, *n* (%)	242 (51.1)	234 (49.8)	0.697	245 (49.5)	251 (56.0)	**0.045**
BMI, mean ± *SD*	24.3 ± 5.6	24.9 ± 5.5	0.144	24.8 ± 5.4	24.4 ± 5.2	0.385
ASA class ≥ III, *n* (%)	30 (6.5)	39 (8.5)	0.256	44 (9.4)	37 (8.4)	0.608
Body temperature ≥ 38 °C, *n* (%)	95 (20.0)	91 (19.4)	0.805	92 (18.6)	100 (22.3)	0.155
CRP, mg/L, mean ± *SD*	72.5 ± 80.1	71.3 ± 76.5	0.821	68.8 ± 70.8	72.3 ± 74.8	0.454
WBC ≥ 11 × 10^9^/L, *n* (%)	359 (75.6)	359 (76.4)	0.772	369 (74.5)	343 (76.6)	0.472
Diagnostic test, *n* (%)						
US	280 (58.9)	326 (69.4)	**0.001**	321 (64.8)	314 (70.1)	0.087
CT	93 (19.6)	41 (8.7)	** < 0.001**	68 (13.7)	32 (7.1)	**0.001**
Multiple imaging studies	93 (19.6)	97 (20.6)	0.685	104 (21.0)	93 (20.8)	0.925
Duration of abdominal pain > 1 day, *n* (%)	183 (38.5)	194 (41.3)	0.388	184 (37.2)	179 (40.0)	0.380
Total length of stay, days, mean ± *SD*	2.2 ± 2.2	2.3 ± 2.2	0.565	2.3 ± 2.7	2.2 ± 2.1	0.422
Postoperative length of stay, days, mean ± sd	2.0 ± 2.2	2.1 ± 2.2	0.976	2.1 ± 2.6	1.9 ± 2.1	0.438
Laparoscopic surgery, *n* (%)	456 (96.2)	447 (95.3)	0.496	490 (99.2)	435 (97.3)	**0.026**
Duration of surgery, minutes, mean ± *SD*	40.7 ± 19.1	40.9 ± 18.3	0.881	43.9 ± 17.4	40.9 ± 17.5	**0.009**
Complex appendicitis, *n* (%)	184 (38.7)	162 (34.7)	0.198	199 (40.3)	161 (36.1)	0.188
Antibiotics use, *n* (%)	172 (36.2)	161 (34.3)	0.529	202 (40.8)	167 (37.3)	0.267
Duration of antibiotics use, days, mean ± *SD*	5.2 ± 3.1	4.8 ± 2.7	0.249	4.3 ± 2.0	4.6 ± 2.8	0.292
Postoperative complications, *n* (%)						
IAA	18 (3.8)	13 (2.8)	0.380	15 (3.0)	15 (3.3)	0.786
SSI	10 (2.1)	9 (1.9)	0.839	9 (1.8)	8 (1.8)	0.967
Clavien-Dindo classification ≥ 3, *n* (%)	8 (29.6)	5 (22.7)	0.586	10 (43.5)	8 (34.8)	0.546
Readmissions, *n* (%)	27 (5.7)	21 (4.5)	0.395	27 (5.5)	19 (4.2)	0.388

^a^First COVID-lockdown: March 12, 2020, to May 31, 2020

^b^First corresponding timeframe 2019: March 12, 2019, to May 31, 2019

^c^Second COVID-lockdown: October 14, 2020, to December 31, 2020

^d^Second corresponding timeframe 2019: October 14, 2019, to December 31, 2019

Numbers may not total 944 and 943 due to missing data

### COVID testing

Of 1854 patients admitted after the onset of COVID-19, 389 were tested for suspected COVID-19 at presentation. Among these patients, 6 patients tested positive. All 6 patients were diagnosed by a PCR test, one patient received an additional COVID antibody test. All patients presented to the hospital within one day after the onset of appendicitis symptoms and all were treated laparoscopically. One patient presented with a complex appendicitis and developed a post-operative infectious complication. The mean post-operative length of stay for the 6 patients was 1.88 days (*SD* ± 2.84).

### Number of performed appendectomies

**Fig. 3:**
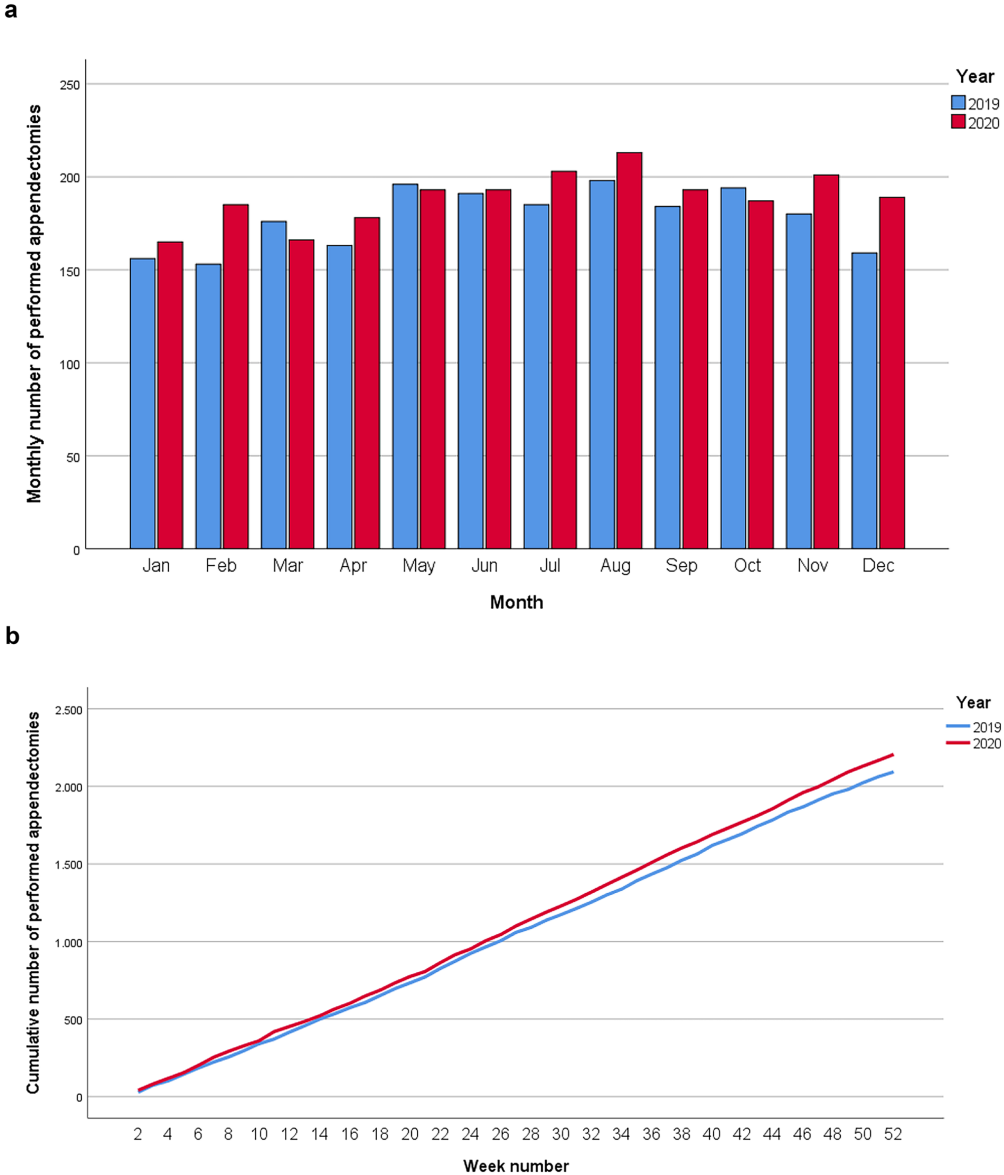
Number of performed appendectomies for acute appendicitis. **a** Monthly number of performed appendectomies, **b** Weekly cumulative number of performed appendectomies

The mean number of performed appendectomies per week during the COVID period (42.1 weeks) was 44.0, compared to 40.9 in the pre-COVID period (62.3 weeks). During the first lockdown (11.6 weeks) and the second lockdown (11.3 weeks), the number of performed appendectomies per week was 40.8 and 43.8, respectively, compared to 40.5 and 39.6 during the equivalent timeframes of 2019. Figure 3 shows number of appendectomies for the years 2019 and 2020.

### Secondary outcomes

In the total study population, 1582 patients had complex appendicitis (35.9%). The proportion of patients with complex appendicitis was comparable for the pre-COVID and COVID group (35.5% vs 36.8%, respectively, *p* = 0.36), as was the proportion of patients presenting to the hospital with a duration of abdominal pain longer than one day (40.6% vs 37.9%, respectively, *p* = 0.07) and the length of postoperative hospital stay in days (2.0 ± 2.2 vs 2.0 ± 2.6, respectively, *p* = 0.93) (Table 1). Similar results were found comparing both lockdowns to their corresponding timeframes of 2019 (Table 2). In total, 159 patients (3.6%) developed IAA within 90 days after appendectomy and 86 patients (2.0%) SSI. No differences were found in the rates of these postoperative infectious complications between the pre-COVID and COVID group (IAA: 3.9% vs 3.2%, respectively, *p* = 0.25 and SSI: 2.0% vs 1.9%, respectively, *p* = 0.79) (Table 1). The subgroup analysis of both COVID-lockdowns showed comparable results (Table 2). The severity of IAA or SSI and readmission rate were not different between the pre-COVID and COVID group and both lockdowns compared to their corresponding timeframes of 2019 (Tables 1 and 2).

### Diagnostic test and surgical approach

US was used in the vast majority of patients. After the onset of COVID-19, the use of a CT was more common than pre-COVID (13.8% vs 9.8%, respectively, *p* < 0.001) (Table 1). US was used significantly less during the first lockdown, compared to the corresponding timeframe of 2019 (58.9% vs 69.4%, *p* = 0.001) (Table 2). During the second lockdown, 490 patients (99.2%) were treated laparoscopically compared to 435 patients (97.3%) during the corresponding timeframe of 2019 (*p* = 0.03). During the first lockdown, the proportion of appendectomies performed laparoscopically was not significantly different from the corresponding timeframe of 2019 (Table 2).

## Discussion and conclusions

In this multicenter study, no difference was observed in the number of appendectomies performed for acute appendicitis during the COVID-19 pandemic, compared to before the pandemic. Furthermore, patient characteristics and treatment outcomes (time between onset of symptoms and hospital admission, proportion of complex appendicitis, total and postoperative length of stay and postoperative complications) remained similar. A shift was observed towards more frequent use of CT and less frequent use of US in the diagnostic pathway during the COVID-19 pandemic.

Other studies have shown a decrease in hospital admissions for acute appendicitis during the COVID-19 pandemic [5–9]. It was hypothesized that patients may have been hesitant to seek medical care due to an aggressive national policy of social distancing and a reduction in public transport [7]. The unchanged presentation and treatment of acute appendicitis in the present study may be explained in several ways. First, to prevent capacity overload in a region or hospital, the National Coordination Center for Patient Distribution coordinated distribution of COVID-19 patients throughout the Netherlands, thereby facilitating continuity of access to healthcare throughout the country, including acute surgical care for acute appendicitis. Secondly, referrals for acute appendicitis may not have decreased due to the relatively mild lockdown measures in the Netherlands. For example, on March 23, 2020, the government advised people to stay at home as much as possible and receive a maximum of three visitors per day. Absolute restrictions of freedom of movement in response to the COVID-19 pandemic were not imposed [23]. Additionally, Google mobility data suggest that during the first six weeks of ‘social distancing’, The Netherlands had one of the highest mobility rates of Europe [24]. Finally, different timeframes of the COVID-19 pandemic were used. For example, Willms et al. included patients with acute appendicitis during a COVID-lockdown period of ten weeks and compared them to patients from the same period in 2019 [8], whereas we included ten months of the COVID pandemic, including a period of diminished restrictions. However, our subgroup analysis of both lockdowns shows comparable outcomes regarding the treatment of acute appendicitis compared to the corresponding timeframes of 2019.

The risk of a simple appendicitis evolving to a complex type may increase with prolonged time between onset of symptoms and hospital referral [25], as was reported in previous studies during the COVID-19 pandemic [8, 10–12, 14]. In contrast to these studies, time between onset of symptoms and hospital admission and the proportion of complex appendicitis remained unchanged in our study population. The observed proportions of complex appendicitis were somewhat high: 35.5% pre-COVID and 36.8% after the onset of COVID. In a multicenter prospective cohort study by Van Rossem et al., the proportion of complex appendicitis in the Netherlands was 29.3% [26]. This difference may be explained by different definitions of complex appendicitis used. We defined complex appendicitis as intraoperative findings of necrosis, perforation, abscess, and/or diffuse peritonitis [21], whereas Van Rossem et al. did not (explicitly) include the presence of an abscess. Furthermore, regional differences might contribute to the high proportion of complex appendicitis. In a study by Giesen et al., which was conducted in the region of Rotterdam, The Netherlands, and included patients from June 2014 to January 2015, a similar proportion of complex appendicitis of 35.3% was found [27].

Scheijmans et al. reported fewer Dutch patients presenting with acute appendicitis during the first COVID-19 lockdown compared to the corresponding timeframe of 2019 [14]. Also, more patients presented with complex appendicitis and more patients were treated conservatively. However, Scheijmans et al. included patients from March 15 to April 30, 2020, and the corresponding timeframe of 2019, whilst the present study included patients over a 2-year interval, comprising both the lockdowns as well as the period in between. Furthermore, Scheijmans et al. included patients from 21 hospitals throughout the Netherlands, while 6 of 9 hospitals in our study are in the region of Rotterdam. The effects of the pandemic may have varied across the country. Moreover, in the region of Rotterdam, hospitals cooperate with each other by transferring patients with, among other, acute appendicitis from one hospital to the next in the event of a capacity overload.

According to the Dutch guidelines, ultrasonography (US) is the first choice imaging modality in the diagnostic pathway of acute appendicitis [28], yet after the onset of COVID-19, an increase in the use of single modality CT was observed, in line with a study by English et al. [17]. It is likely that a CT was preferred over US more often during the COVID-outbreak as a means to reduce direct patient contact. Despite the exposure of patients to ionizing radiation, the use of a CT as first-line imaging for evaluating acute abdominal pain has been advocated before, given its higher accuracy compared to US [29]. Moreover, Haijanen et al. reported the use of low dose ionizing radiation in CT as still accurate in identifying an acute appendicitis [30].

This study focused solely on the surgical treatment of acute appendicitis, selecting patients by use of hospital registry codes for appendectomy. Therefore, no firm conclusion can be drawn concerning the frequency of non-operative treatment. However, De Wijkerslooth et al. demonstrated a yearly decrease in the incidence of acute appendicitis [31]. Given the similar number of performed appendectomies in 2020 compared to 2019 in our study, it is less likely that a conservative treatment of acute appendicitis was implemented on a wider scale. Some studies demonstrated an increase in open surgery to avoid the potential risk of aerosolization [17, 19]. In contrast, we observed a significantly higher rate of laparoscopy during the second lockdown. Moreover, all six COVID-19 positive patients were treated laparoscopically. This may be explained by the fact that the Dutch Association of Surgeons did not explicitly propagate open surgery over laparoscopy in response to the pandemic [20].

The main strength of this study is its multicenter design, resulting in a large number of patients. To our knowledge, no other large-scale study has presented results for a patient population including the first as well as the second COVID-19 wave. The retrospective design is its main limitation as there remains an extent of missing data, most markedly in BMI and ASA classification. Furthermore, some eligible patients may have been missed due to inaccurate registration of reimbursement/procedure codes.

In conclusion, in this study, surgical care and outcomes for patients with acute appendicitis in 9 hospitals in the Netherlands seem unaffected by the outbreak of COVID-19 and the subsequent pressure on the healthcare system. Distribution of COVID-19 patients throughout the country and solid (regional) cooperation between hospitals are key in healthcare continuity in times of a pandemic.
